# Oral magnesium supplementation does not affect insulin sensitivity in people with insulin-treated type 2 diabetes and a low serum magnesium: a randomised controlled trial

**DOI:** 10.1007/s00125-023-06029-9

**Published:** 2023-11-03

**Authors:** Linda C. A. Drenthen, Jeroen H. F. de Baaij, Laura Rodwell, Antonius E. van Herwaarden, Cees J. Tack, Bastiaan E. de Galan

**Affiliations:** 1 Department of Internal Medicine, Radboudumc, Nijmegen, the Netherlands; 2 Department of Medical Biosciences, Radboudumc, Nijmegen, the Netherlands; 3 Department for Health Evidence, Section Biostatistics, Radboudumc, Nijmegen, the Netherlands; 4 Department of Laboratory Medicine, Radboudumc, Nijmegen, the Netherlands; 5 Department of Internal Medicine, Maastricht University Medical Center+ (MUMC+), Maastricht, the Netherlands

**Keywords:** Euglycaemic clamp, Insulin resistance, Insulin sensitivity, Magnesium supplementation, Type 2 diabetes mellitus

## Abstract

**Aims/hypothesis:**

Hypomagnesaemia has been associated with insulin resistance and an increased risk of type 2 diabetes. Whether magnesium supplementation improves insulin sensitivity in people with type 2 diabetes and a low serum magnesium level is unknown.

**Methods:**

Using a randomised, double-blind (both participants and investigators were blinded to the participants’ treatment sequences), placebo-controlled, crossover study design, we compared the effect of oral magnesium supplementation (15 mmol/day) for 6 weeks with that of matched placebo in individuals with insulin-treated type 2 diabetes (age ≥18 years, BMI 18–40 kg/m^2^, HbA_1c_ <100 mmol/mol [11.3%], serum magnesium ≤0.79 mmol/l). Participants were recruited from the outpatient clinic and through advertisements. Randomisation to a treatment sequence order was done using a randomisation list. We used block randomisation and the two possible treatment sequences were evenly distributed among the trial population. The primary outcome was the mean glucose infusion rate during the final 30 min of a hyperinsulinaemic–euglycaemic clamp (i.e. *M* value). Secondary outcomes included variables of glucose control, insulin need, BP, lipid profile and hypomagnesaemia-related symptoms during follow-up.

**Results:**

We recruited 14 participants (50% women, 100% White, mean ± SD age 67±6 years, BMI 31±5 kg/m^2^, HbA_1c_ 58±9 mmol/mol [7.4±0.9%]) with insulin-treated type 2 diabetes. Magnesium supplementation increased both mean ± SEM serum magnesium level (0.75±0.02 vs 0.70±0.02 mmol/l, *p*=0.016) and urinary magnesium excretion (magnesium/creatinine ratio, 0.23±0.02 vs 0.15±0.02, *p*=0.005), as compared with placebo. The *M* value of the glucose clamp did not differ between the magnesium and placebo study arms (4.6±0.5 vs 4.4±0.6 mg kg^−1^ min^−1^, *p*=0.108). During the 6 weeks of treatment, continuous glucose monitoring outcomes, HbA_1c_, insulin dose, lipid profile and BP also did not differ, except for a lower HDL-cholesterol concentration after magnesium compared with placebo (1.14±0.08 vs 1.20±0.09 mmol/l, *p*=0.026). Symptoms potentially related to hypomagnesaemia were similar for both treatment arms.

**Conclusions/interpretation:**

Despite an albeit modest increase in serum magnesium concentration, oral magnesium supplementation does not improve insulin sensitivity in people with insulin-treated type 2 diabetes and low magnesium levels.

**Trial registration:**

EudraCT number 2021-001243-27.

**Funding:**

This study was supported by a grant from the Dutch Diabetes Research Foundation (2017–81–014).

**Graphical Abstract:**

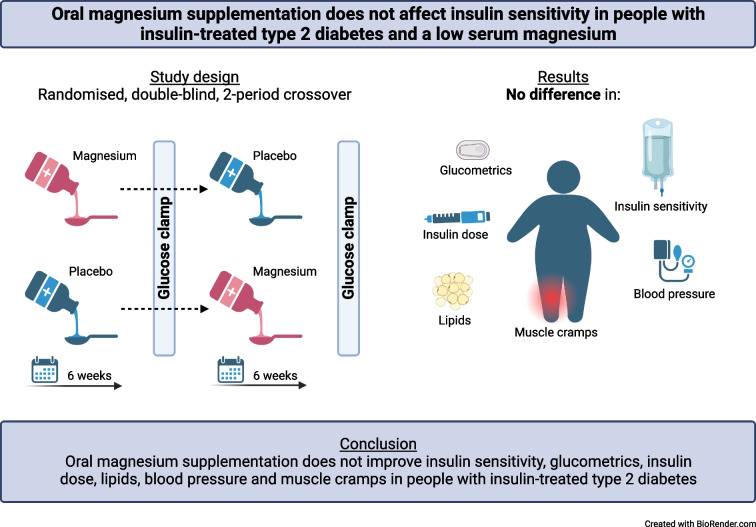

**Supplementary Information:**

The online version of this article (10.1007/s00125-023-06029-9) contains peer-reviewed but unedited supplementary material.


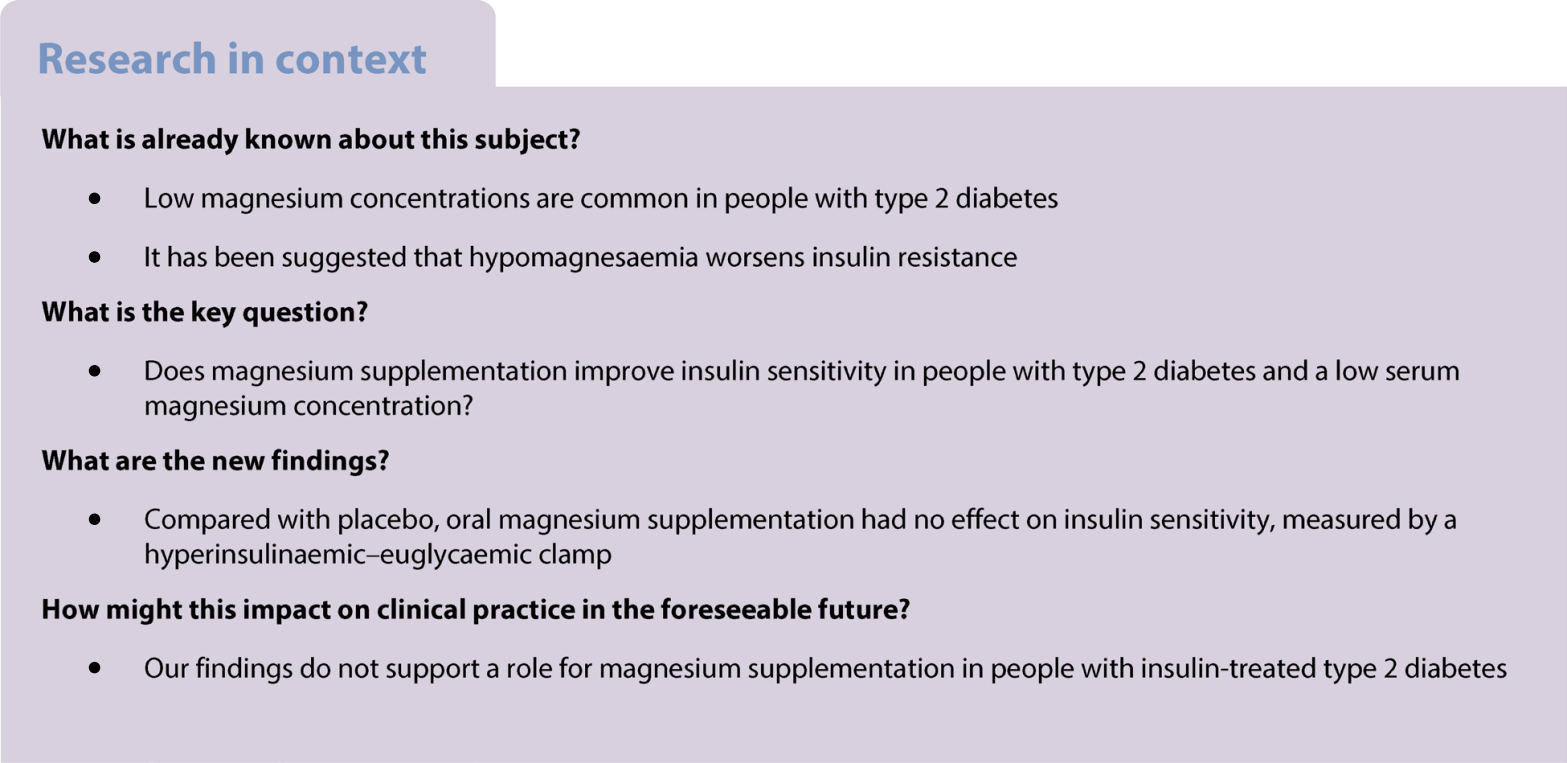


## Introduction

Low serum magnesium has been implicated in the pathogenesis of type 2 diabetes and its cardiovascular complications [1]. Previous studies have shown that hypomagnesaemia is prevalent in 9–48% of people with type 2 diabetes [2–4] and the dietary intake of magnesium is inversely associated with the incidence of type 2 diabetes [5–7]. In people with type 2 diabetes, insulin secretion is progressively lost and insufficient to compensate for insulin resistance, the latter being a cardinal feature of type 2 diabetes [8]. We have previously demonstrated that pancreatic beta cell action is not affected by magnesium [9], yet the level of insulin resistance may be worsened by magnesium deficiency. Although the molecular mechanisms underlying the effect of magnesium on insulin sensitivity are not fully understood, most evidence points to a reduced autophosphorylation of the insulin receptor, increased chronic inflammatory state, and reduced GLUT-4 expression associated with hypomagnesaemia [4, 10–13].

Administering a single i.v. dose of magnesium improves insulin sensitivity in people with stress-induced hyperglycaemia [14]. Using the euglycaemic clamp technique, Paolisso et al showed that magnesium supplementation improved insulin sensitivity in people with type 2 diabetes who are treated with diet or oral medication and had a normal serum magnesium concentration [15]. More recent meta-analyses show conflicting results regarding the effect of magnesium supplementation on insulin sensitivity, fasting glucose and HbA_1c_ in people with type 2 diabetes [16–18]. However, most studies applied HOMA to assess insulin sensitivity rather than the euglycaemic clamp technique, which is viewed as the gold standard for measuring insulin sensitivity. Moreover, participants were usually neither treated with insulin nor selected for low magnesium levels and most studies date from 20 or more years ago while studies performed in contemporary cohorts are lacking.

Thus, it is currently unclear whether magnesium supplementation can improve insulin sensitivity in people with type 2 diabetes and a low magnesium concentration. Low magnesium levels seem most prevalent in advanced diabetes, and people with this condition are often on insulin treatment [3]. As this group would be expected to derive the greatest benefit from improvements in insulin sensitivity, we studied people with insulin-treated type 2 diabetes and a low magnesium concentration and examined the effect of magnesium supplementation on insulin resistance using the euglycaemic clamp technique.

## Methods

### Study design

This was a randomised, double-blind (both participants and investigators were blinded to the participants’ treatment sequences), placebo-controlled, two-period, crossover intervention study performed at the Radboudumc (Nijmegen, the Netherlands). The study was approved by the local ethics committee and national competent authority and conducted in accordance with the principles of the Declaration of Helsinki, the Medical Research Involving Human Subjects Act, and applicable ICH Good Clinical Practice guidelines. All participants provided written informed consent.

### Study population

Participants were recruited from the outpatient clinic of the Radboudumc and through websites of patient associations. Adults (aged ≥18 years) with type 2 diabetes were eligible for participation when they were treated with insulin for at least 1 year and had a serum magnesium concentration ≤0.79 mmol/l. Key exclusion criteria were HbA_1c_ >100 mmol/mol (>11.3%), a total daily insulin dose of >2 U/kg body weight, MDRD-GFR <45 ml/min per 1.73 m^−2^, BMI <18 or >40 kg/m^2^, pregnancy, chronic diarrhoea and self-reported alcohol consumption >14 units weekly. People using magnesium supplements were not excluded from participation provided that they agreed to stop these at least 1 week before screening. Participant enrolment ran from March to August 2022 and we believe that our study sample is representative of the larger population of interest. Sex/gender and race/ethnicity were self-reported.

### Study procedure

Participants first came for a screening visit, which included medical history, standard physical examination and BP measurement. Blood was sampled for determination of HbA_1c_, potassium, calcium, albumin, lipid profile and creatinine. Urine was sampled for determination of magnesium and creatinine.

**Fig. 1:**
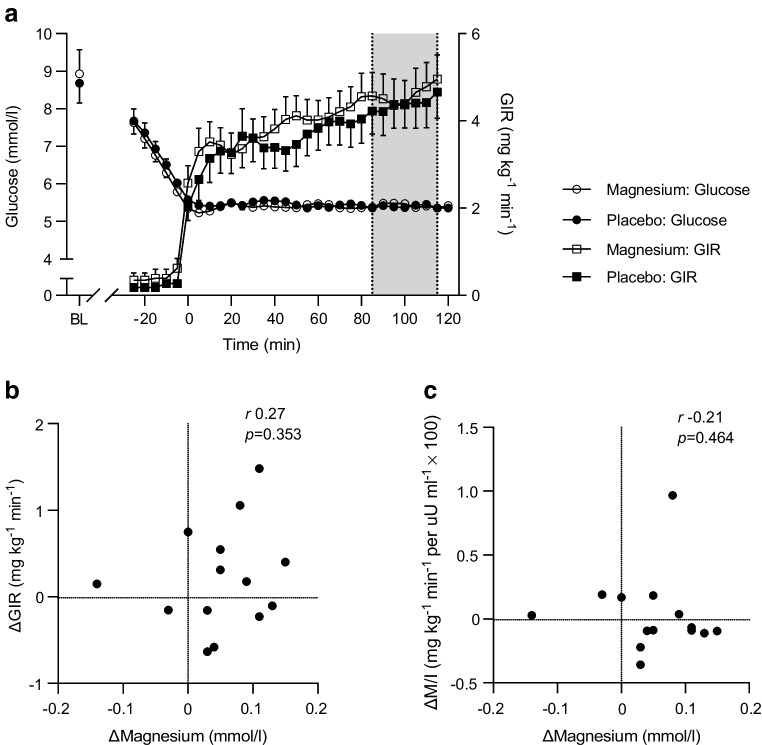
Time course of the plasma glucose concentration and GIR during the hyperinsulinaemic–euglycaemic clamp (**a**); the correlation between the change in serum magnesium concentration and the change in mean GIR (**b**); and the correlation between the change in serum magnesium concentration and the change in mean insulin sensitivity index (M/I) after magnesium supplementation compared with placebo (**c**). The grey area in (**a**) represents the last 30 min of the glucose clamp. Data are presented as mean ± SEM (*n*=14) (**a**) or individual values (**b**, **c**). BL, baseline

After inclusion, participants were randomly assigned to treatment with magnesium gluconate or matching placebo for 6 weeks in a crossover design (electronic supplementary material [ESM] Fig. 1). Randomisation was done using a randomisation list (sequentially numbered) and performed by the pharmacy department, to ensure the double-blind nature of the study. The two treatment sequences were evenly distributed among the trial population using block randomisation. Magnesium gluconate and placebo were administered orally as a liquid solution of 50 ml three times a day, resulting in a daily dose of 150 ml (15 mmol magnesium, equivalent to 360 mg). Possible dose-related side-effects were monitored weekly by telephone consultation and the dose could be reduced step by step if necessary. If participants did not tolerate a minimum dose of 50 ml daily, they were withdrawn from study participation. During the final week of each treatment period, participants wore a blinded continuous glucose monitoring (CGM) device (Dexcom G6; Dexcom, San Diego, CA, USA) and activity tracker (activPAL micro; PAL technologies, Glasgow, UK) for 7 days [19]. On the day CGM and the activity tracker started, each participant’s BP was measured for 30 min using automated BP monitoring.

At the end of each treatment period of 6 weeks, participants underwent a hyperinsulinaemic–euglycaemic clamp (target glucose 5.5 mmol/l) [20]. Briefly, participants attended the research facility at 08:00 hours after an overnight fast, having abstained from alcohol, caffeine and smoking for 24 h and from strenuous exercise for 48 h. They were asked to interrupt or reduce their long-acting insulin dose 24 h before the clamp and omit their morning insulin dose and other glucose-lowering medication if applicable. After arrival, body weight was measured and the participants filled out a questionnaire that assessed potential symptoms of hypomagnesaemia [1, 21, 22] (ESM Methods). These potential symptoms included: muscle cramps, myalgia, muscle weakness, stiffness, tingling, restless legs, palpitations, fatigue, sleeping problems and difficulty concentrating. A urine sample was collected for measuring magnesium and creatinine. One catheter was inserted in retrograde fashion into a forearm vein for frequent blood sampling and this forearm was placed in a heated box (~55°C) to arterialise venous blood. Another catheter was placed into the antecubital vein of the contralateral arm for infusion of glucose 20% (wt/vol.; Baxter, Deerfield, IL, USA) and insulin (insulin aspart; Novo Nordisk, Bagsvaerd, Denmark). Baseline hyperglycaemia was corrected as needed with a mean i.v. bolus of 4 U of insulin (range 0–16 U). Subsequently, insulin was administered at a stable dose of 120 mU m^−2^ min^−1^, which is likely to fully suppress hepatic glucose production, and glucose 20% was administered at a variable dose to obtain stable euglycaemia (target glucose concentration of 5.5 mmol/l for 120 min), based on plasma glucose measured every 5 min using Biosen C-Line (EKF Diagnostics, Cardiff, UK). At baseline, blood was sampled for determination of HbA_1c_, magnesium, potassium, calcium, albumin, lipid profile, creatinine and insulin. After 90 and 120 min, blood was sampled to measure insulin.

### Study outcomes

The primary outcome was the mean glucose infusion rate (GIR) during the final 30 min of the clamp (i.e. *M* value) [23]. Secondary outcomes included the insulin sensitivity index, calculated as the *M* value divided by the insulin concentration (M/I), mean daily insulin dose administered on the 3 days before the clamp, HbA_1c_, hypomagnesaemia-related symptoms determined by the questionnaire, mean BP measured for 30 min using automated BP monitoring and lipid profile, as well as CGM and activity tracker outcomes. CGM outcomes included mean glucose concentration, CV (i.e. SD divided by mean glucose concentration), occurrence of hypoglycaemic events, and time spent above range (i.e. glucose >10.0 mmol/l) and in range (i.e. glucose ≥3.9 and ≤10.0 mmol/l), as derived from CGM downloads. A hypoglycaemic event was defined as a glucose concentration <3.9 mmol/l for at least 15 consecutive minutes and a new event was calculated if the glucose concentration had been above this level for at least 15 min [24]. All CGM outcomes were calculated using R version 4.1.2 (R Foundation for Statistical Computing, Vienna, Austria). All raw activity tracker data were analysed by a modified version of the previously developed script of Winkler et al [25], using SAS version 9.4 (SAS Institute, NC, USA), and included sitting time, active time (i.e. standing and stepping) and number of steps. Physical activity intensity was classified as light (i.e. metabolic equivalent of task [MET] score <3) or moderate-to-vigorous (i.e. MET-score ≥3) [26].

### Measurements

HbA_1c_ was measured in EDTA whole blood on a Tosoh G11 in variant HbA_1c_ mode with ion-exchange chromatography and absorbance detection (Sysmex, the Netherlands). Magnesium, lipid profile, calcium, albumin (bromocresol purple), creatinine (enzymatic) and potassium (ion-selective electrode) in plasma (and urine) were measured on a random access analyser Cobas 8000 (Roche Diagnostics, the Netherlands). Plasma insulin was measured using an in-house radioimmunoassay, using guinea pig anti-human insulin antibody and ^125^I-labelled human insulin tracer. In this assay, calibrated on WHO international standard 83/500, bound–free separation was performed by second antibody/polyethylene glycol precipitation of antibody-bound insulin [27].

### Statistical analysis

To identify an increase of at least 20% in GIR after magnesium supplementation with a statistical power of 80% [15], we calculated that 14 participants would be required to detect a difference at a significance level of 0.05. We used random effects models to account for the two measurements for each participant with period and treatment as independent variables. Continuous variables were analysed using a multilevel mixed-effects linear regression model performing restricted maximum-likelihood estimation, and binary outcomes were analysed using a logistic random effects model. Data that were not normally distributed were log transformed. We performed a sensitivity analysis for data that were near normally distributed using the Wilcoxon signed rank test, which showed similar results to the mixed model. Correlations were analysed using Spearman’s rank-order test. No adjustments were made to account for multiple testing. Data were analysed using SPSS version 27 (IBM, NY, USA) and Stata version 17 (StataCorp, TX, USA). All data are expressed as mean ± SEM, unless otherwise specified. A *p* value <0.05 was considered statistically significant.

## Results

A total of 30 potentially eligible participants were screened, 16 of whom were excluded because their serum magnesium concentration was too high (*n*=14) or for other reasons (*n*=2) (ESM Fig. 2). All 14 included participants completed the study. Their baseline characteristics are shown in Table 1. A flash glucose monitoring device was used by eight participants (57%), five of whom had the alarm function for low and high glucose concentrations turned on. Four participants (29%) were on magnesium supplementation before the study but stopped using this at least 1 week before the screening visit. No serious adverse events occurred during the study. Two participants had a COVID-19 infection, one was diagnosed with gout, one had a mild mycosis on his foot, and one was diagnosed with occipital neuralgia, none of which were judged to be study-related or to impact on the study results.

**Table 1:** Baseline characteristics

Characteristic	
No. of participants	14
Age, years	67±6
Female sex	7 (50)
White race	14 (100)
BMI, kg/m^2^	31.3±4.9
Duration of diabetes, years	19±8
HbA_1c_, mmol/mol	58±9
HbA_1c_, %	7.4±0.9
Glucose-lowering treatment	
Long-acting insulin	13 (93)
Short-acting insulin	10 (71)
Metformin	12 (86)
Sulfonylureas	4 (29)
GLP-1 analogues	2 (14)
SGLT2 inhibitors	1 (7)
Duration of insulin use, years	13±9
Total insulin dose, U/day	55±26
Microvascular complications	
Retinopathy	3 (21)
Nephropathy	3 (21)
Neuropathy	3 (21)
Serum magnesium, mmol/l	0.73±0.05
Serum creatinine, µmol/l	81±20
Serum triglycerides, mmol/l	2.3±1.3
Systolic BP, mmHg	139±13
Diastolic BP, mmHg	71±10

Data are presented as mean ± SD or *n* (%)

GLP-1, glucagon-like peptide 1; SGLT2, sodium–glucose cotransporter 2

### General data

The total amount of study medication taken by the participants was similar during the magnesium and placebo treatment periods (5610±208 vs 5637±161 ml, *p*=0.844; both 89±3% of target). Serum magnesium level was higher after magnesium supplementation compared with placebo (0.75±0.02 vs 0.70±0.02 mmol/l, *p*=0.016); this was also the case for the urinary magnesium excretion (magnesium/creatinine ratio 0.23±0.02 vs 0.15±0.02, *p*=0.005). Serum potassium (4.1±0.1 vs 4.0±0.1 mmol/l, *p*=0.642) and calcium concentrations (corrected for serum albumin 2.4±0.0 vs 2.4±0.0 mmol/l, *p*=0.960) were similar after magnesium compared with placebo, as was body weight (92.5±3.9 vs 93.2±4.1 kg, *p*=0.142) (Table 2). Reported side-effects were change in stool pattern (88%), nausea (38%) and flatulence (19%), which occurred in four participants on magnesium treatment, in four participants on placebo treatment and in four participants on both magnesium and placebo treatment. A dose reduction to 50 ml twice daily was required for one participant during magnesium treatment.

**Table 2:** Body weight, glucose control, CGM outcomes, lipids, BP and physical activity variables after magnesium and placebo treatment

Variable	Magnesium	Placebo	*p* value
General characteristics			
Weight, kg	92.5±3.9	93.2±4.1	0.142
BMI, kg/m^2^	30.9±1.2	31.1±1.3	0.160
Glucose control			
HbA_1c_, mmol/mol	57±2	58±2	0.851
HbA_1c_, %	7.4±0.2	7.4±0.2	
Insulin dose, U/day^a^	44±6	44±7	0.869
CGM^a^			
Mean glucose concentration, mmol/l	9.4±0.5	9.4±0.4	0.920
CV, %	26±1	27±2	0.247
Hypoglycaemic events, yes	5 (36)	4 (29)	0.558
Time in range, min	956±91	937±76	0.832
Time above range, min	470±88	495±76	0.773
Lipids			
Total cholesterol, mmol/l	4.3±0.2	4.2±0.3	0.483
Triglycerides, mmol/l	2.3±0.5	1.8±0.3	0.261
LDL, mmol/l^a^	2.1±0.2	2.1± 0.2	0.627
HDL, mmol/l	1.14±0.08	1.20±0.09	0.026
BP			
Systolic BP, mmHg	135±3	135±5	0.916
Diastolic BP, mmHg	68±2	68±3	0.817
Physical activity^b^			
Sitting time, h	10.5±0.3	10.2±0.4	0.219
Active time, h	4.2±0.4	4.7±0.5	0.098
LIPA, min	204±19	234±29	0.085
MVPA, min	47±7	46±6	0.714
Step count, *n*	6038±827	6007±746	0.925

Data are presented as mean ± SEM or *n* (%)

Hypoglycaemic events are expressed as a binary outcome. Other CGM and physical activity data are expressed as mean per day

^a^*n*=12

^b^*n*=13

LIPA, light physical activity; MVPA, moderate-to-vigorous physical activity

### Clamp data

The clamps after the two treatment periods did not differ with regard to the total number of units of insulin administered during the clamp (magnesium vs placebo, 44±2 vs 42±2 units, *p*=0.426) nor for the time between start of insulin infusion and T=0 (both 43±5 min, *p*=0.936). Furthermore, during the last 30 min of the clamp, mean glucose concentration (5.4±0.02 vs 5.4±0.01 mmol/l, *p*=0.212), CV (3.0±0.2 vs 2.8±0.2%, *p*=0.420) and steady-state insulin concentration (3952±498 vs 3578±441 pmol/l, *p*=0.113) were similar for the magnesium and placebo study arms. The mean GIR during the final 30 min of the clamp did not differ after magnesium compared with placebo (4.6±0.5 vs 4.4±0.6 mg kg^−1^ min^−1^, *p*=0.108) (Fig. 1a). Furthermore, there were no differences for M/I between the study arms (magnesium vs placebo 1.1±0.2 vs 1.0±0.2 mg kg^−1^ min^−1^ per µU/ml × 100, *p*=0.675). We found no correlation between the change in magnesium and either change in GIR or change in M/I after treatment with magnesium (Fig. 1b,c).

### Glucose control, lipids and BP

No differences in HbA_1c_, CGM outcomes, insulin dose or BP were found between the two study arms (Table 2). HDL-cholesterol was significantly lower after magnesium compared with placebo (1.14±0.08 vs 1.20±0.09 mmol/l, *p*=0.026), but no effect on total cholesterol, LDL-cholesterol and triglycerides was found (Table 2).

### Hypomagnesaemia-related symptoms and physical activity

**Fig. 2:**
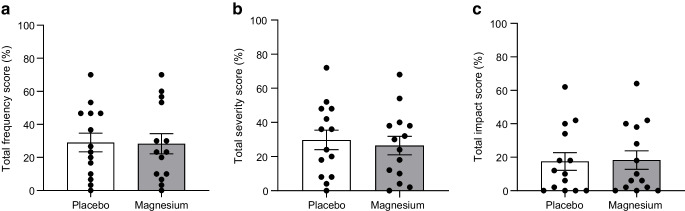
Percentage of total score on questionnaire regarding frequency (**a**), severity (**b**) and daily-life impact (**c**) of hypomagnesaemia-related symptoms. Data are presented as individual values; bars represent mean ± SEM

There was no difference in frequency or severity of hypomagnesaemia-related symptoms after magnesium compared with placebo (Fig. 2). Additionally, we found no correlation between the change in magnesium and change in symptom frequency or severity (ESM Fig. 3). No difference in physical activity was present between the two study arms (Table 2).

## Discussion

The main finding of this study is that oral magnesium supplementation, despite significantly increasing the serum magnesium concentration, did not improve insulin sensitivity in people with insulin-treated type 2 diabetes and low magnesium levels. Furthermore, during the 6 week treatment periods, magnesium supplementation did not improve variables of glucose control, insulin need, lipid profile or BP and had no effect on hypomagnesaemia-related symptoms. These results do not support magnesium supplementation to people with insulin-treated type 2 diabetes and a low serum magnesium concentration.

Oral magnesium supplementation increased serum magnesium by a mean of 0.05 mmol/l in our study participants, which is in line with previous reports [4, 28]. We used magnesium gluconate because organic magnesium salts have a higher bioavailability than inorganic magnesium salts [29], as shown in magnesium-depleted rats [30]. Furthermore, we used multi-day administration rather than single dosing to optimise intestinal uptake of magnesium [30]. The participants’ adherence to study medication in our study was excellent. Some of the participants used medication that can cause hypomagnesaemia (e.g. thiazide diuretics [*n*=5] or proton pump inhibitors [*n*=12]) but their dose did not change during the study period and previous research has shown that less than 10% of hypomagnesaemia is attributable to medication use in people with type 2 diabetes [3]. While serum magnesium levels increased, we also found that the urinary magnesium excretion increased after magnesium supplementation, possibly suggesting a threshold. It is therefore questionable whether a higher magnesium dose could have further increased serum magnesium in this population, even when tolerated given the increased risk for (gastrointestinal) side-effects associated with higher doses.

While we can only compare the insulin sensitivity between the two study arms, comparison with literature [31, 32] shows that the study participants indeed had a low GIR, as expected and consistent with the presence of insulin resistance. Clinical trials studying the effect of magnesium supplementation on insulin sensitivity have shown conflicting results [16–18]. Our findings contrast with those of a study using the glucose clamp technique to report an increase in insulin sensitivity after magnesium supplementation in people with type 2 diabetes [15]. Although the dose of magnesium used for supplementation and the increase in magnesium levels were comparable, the participants of that study were treated with diet or oral medication alone and had higher baseline magnesium levels, making the clinical relevance of their findings questionable. Two other studies reported no effect of magnesium supplementation on insulin sensitivity in people with insulin-treated type 2 diabetes, again with normal magnesium levels, yet using HOMA as a measure for insulin sensitivity [33, 34]. Our results extend these findings to a population selected by a low serum magnesium level and provide validation by using the gold standard for measuring insulin sensitivity.

In our study, magnesium supplementation did not improve glucose control, which is in line with unaltered insulin sensitivity and insulin dose. The study duration may have been too short to show an effect on HbA_1c_ but the CGM outcomes did not differ between the study arms. We are the first to explore the effect of magnesium on CGM outcomes in people with type 2 diabetes. Previous studies have shown conflicting results regarding the effect of magnesium on HbA_1c_ and fasting glucose, with most trials having been performed among participants treated with diet or oral medication alone [16–18]. In line with our results, de Valk et al showed that magnesium supplementation for 3 months did not improve fasting glucose or HbA_1c_ in people with insulin-treated type 2 diabetes [35].

Previous studies reported improvements in BP and lipids after magnesium supplementation but our study might have been too short and underpowered for these outcomes [36–38]. Our results showed, surprisingly, that HDL-cholesterol slightly decreased after magnesium supplementation, although we have to interpret this result with caution, since we did not correct for multiple testing within our analyses. Moreover, because the mean HDL-cholesterol concentration hardly differed between the study arms, one can question the clinical relevance of this finding.

In exploratory analyses, we found that despite strong beliefs, hypomagnesaemia-related symptoms were similar after magnesium and placebo treatment and there was no correlation between the change in magnesium level and change in symptom frequency and severity. Low potassium and calcium levels produce symptoms similar to those of hypomagnesaemia and are often simultaneously present, but both were within normal ranges in our participants. Muscle cramps are the most abundant symptom caused by hypomagnesaemia but meta-analyses data show inconsistent results regarding the effect of magnesium supplementation on muscle cramps in people not necessarily selected for a low magnesium concentration [39, 40].

Strengths of our study are the randomised, placebo-controlled, double-blind, crossover design, the use of the glucose clamp technique, which is the gold standard for measuring insulin sensitivity, and the inclusion of a study population that was found to be clearly insulin resistant and therefore most likely to benefit to the greatest extent from improvements in insulin sensitivity.

Our study also has limitations. First, we have no registration of the diet of the participants, although we do not expect a change in eating habits during the study period, especially because the occurrence of gastrointestinal side-effects was evenly distributed among the two treatments. Besides, dietary records often include bias and because we found an increase in serum magnesium after supplementation, reporting dietary magnesium intake becomes less important. Furthermore, variables potentially affecting insulin sensitivity such as body weight, physical activity and glucose control, all objectively measured, did not differ between the study arms. Second, while magnesium levels increased significantly, the increase was relatively modest and participants remained at the low end of the normal range. Although serum magnesium is generally accepted to reflect the overall magnesium status, we have no information on the intracellular magnesium concentration. Our study does not exclude the possibility that supplementation to higher magnesium levels could have a positive effect on insulin sensitivity in this population. However, as discussed above, oral supplementation to higher levels may not be possible due to adverse effects with higher dosing, including diarrhoea that may promote intestinal loss of magnesium, and the possible concomitant greater urinary excretion of magnesium. Third, we cannot generalise our results to people with type 2 diabetes and poorer glucose control or to those with a higher baseline serum magnesium concentration, as is the case for the majority of people with type 2 diabetes [2–4]. However, since plasma magnesium is negatively correlated with insulin resistance [13], we expect no improvement in insulin sensitivity after magnesium supplementation in people with insulin-treated type 2 diabetes and a normal serum magnesium. Furthermore, although we performed a sample size calculation, the sample size was relatively small, possibly suggesting that our study was underpowered. However, for our findings to become statistically significant, we would have required such a large study population that the clinical relevance of the study would be questionable. Finally, using a crossover study design can have disadvantages, including carry-over effects. We would not expect a period effect to be present but additionally minimised this risk by adjusting for it within our statistical analyses. Since the placebo study arm already served as a wash-out period and we expect the blood magnesium concentration to reach a new steady state within a couple of days after discontinuation of supplementation [41], we did not include an additional wash-out period with the advantage that participants were not interrupted in their routine of taking the study medication.

In conclusion, oral magnesium supplementation does not improve insulin sensitivity in people with insulin-treated type 2 diabetes and a low magnesium level, neither does it affect glucose control, lipid profile, BP and hypomagnesaemia-related symptoms. Therefore, our trial does not support routine use of magnesium supplementation in people with insulin-treated type 2 diabetes.

### Supplementary Information

Below is the link to the electronic supplementary material.

Supplementary file1 (PDF 253 KB)

## Data Availability

The datasets generated during and/or analysed in the current study are available from the corresponding author upon reasonable request.

## Abbreviations

CGM: Continuous glucose monitoring
GIR: Glucose infusion rate
MET: Metabolic equivalent of task
M/I: Insulin sensitivity index (*M* value divided by the insulin concentration)
