# Supplementary material for: Oral magnesium supplementation does not affect insulin sensitivity in people with insulin-treated type 2 diabetes and a low serum magnesium: a randomised controlled trial
Source: Diabetologia. 2023 Nov 3;67(1):52–61. doi: 10.1007/s00125-023-06029-9 (PMC10709477; doi:10.1007/s00125-023-06029-9)
Supplement: Supplementary file 1 — Supplementary file1 (PDF 253 KB) [file 125_2023_6029_MOESM1_ESM.pdf]

Electronic supplementary material (ESM)

ESM Methods: Questionnaire symptoms magnesium

Question 1: In the past three weeks, how often have you suffered from:

	Daily<br><br>*(3 points)*	Several days a week<br>*(2 points)*	Once a week or less<br>*(1 point)*	Not at all<br><br>*(0 points)*
Muscle cramps				
Myalgia				
Muscle weakness				
Stiffness				
Tingling				
Restless legs				
Palpitations				
Fatigue				
Sleeping problems				
Difficulty concentrating				

Maximum score: 30

Question 2: In the past three weeks, to what extent have you suffered from:

	Very much<br><br>*(5 points)*	Quite a lot<br>*(4 points)*	Somewhat<br><br>*(3 points)*	A little<br><br>*(2 points)*	Almost not<br>*(1 point)*	Not at all<br><br>*(0 points)*
Muscle cramps						
Myalgia						
Muscle weakness						
Stiffness						
Tingling						
Restless legs						
Palpitations						
Fatigue						
Sleeping problems						
Difficulty concentrating						

Maximum score: 50

Question 3: In the past three weeks, to what extent were you limited in your daily activities by:

	Very much *(5 points)*	Quite a lot *(4 points)*	Somewhat *(3 points)*	A little *(2 points)*	Almost not *(1 point)*	Not at all *(0 points)*
Muscle cramps						
Myalgia						
Muscle weakness						
Stiffness						
Tingling						
Restless legs						
Palpitations						
Fatigue						
Sleeping problems						
Difficulty concentrating						

Maximum score: 50

ESM figures

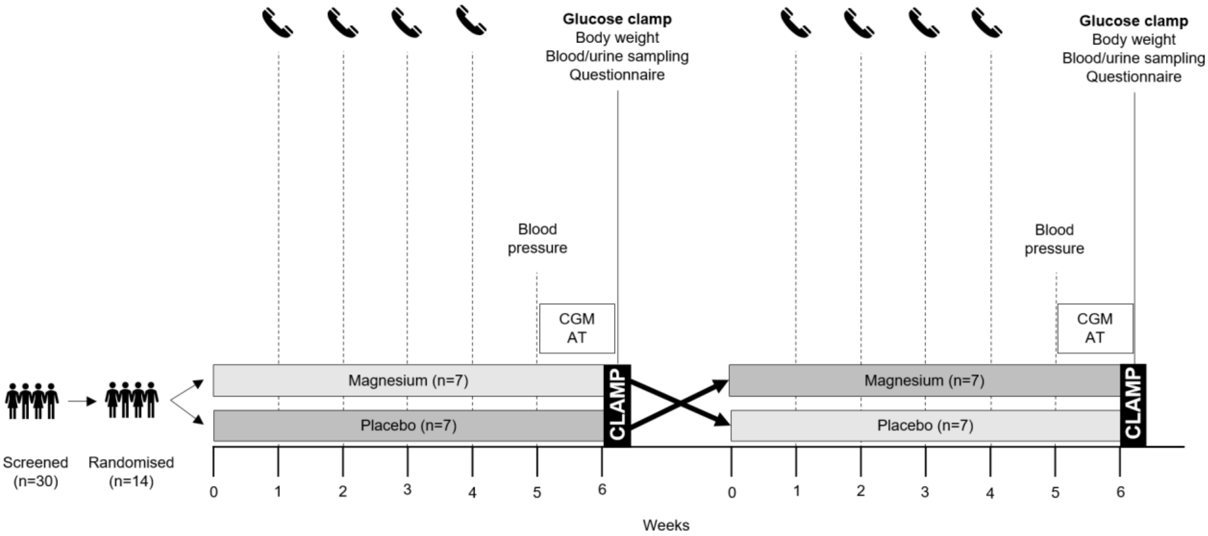

ESM Fig. 1 – Study design. CGM = continuous glucose monitoring; AT = activity tracker.

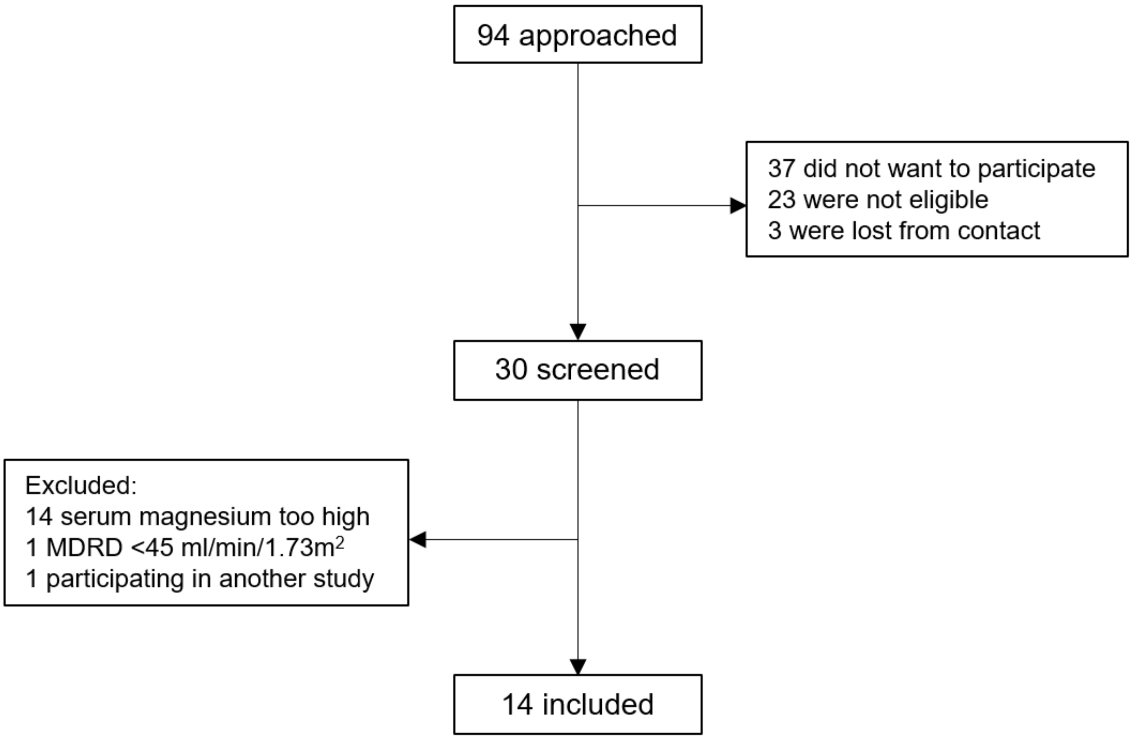

ESM Fig. 2 – Flowchart.

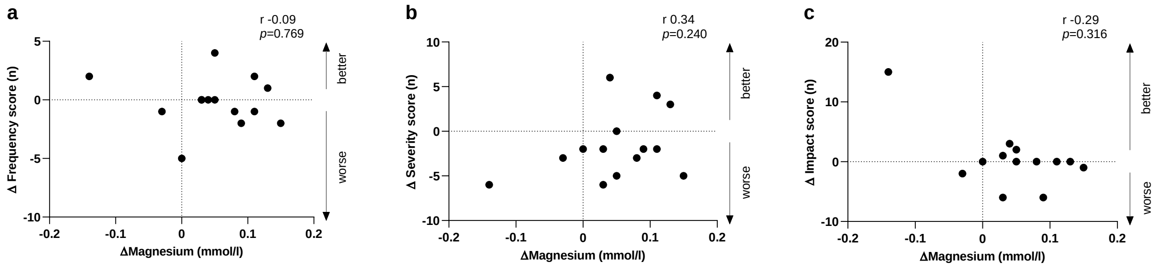

ESM Fig. 3 – Correlation between the change in serum magnesium concentration and the change in frequency (a), severity (b), and daily-life impact (c) score of hypomagnesemia-related symptoms after magnesium compared to placebo treatment. Data are presented as individual values.
